# Exploring trauma surgeons' views on trauma care in Nigeria: A qualitative study

**DOI:** 10.1016/j.afjem.2024.03.001

**Published:** 2024-07-14

**Authors:** Oluwafunmilayo Akinlade, Adebisi Adeyeye, Brandon L. Ellsworth, Christopher W. Reynolds, Chiamaka Eneh, Ayobami Olufadeji

**Affiliations:** a Department of Emergency Medicine, University of Virginia Health System, Charlottesville, VA, USA; b College of Medicine, University of Lagos, Lagos, Nigeria; c Department of Internal Medicine, Northwestern Medicine, Chicago, IL, USA; d University of Michigan Medical School, Ann Arbor, MI, USA; e Department of Emergency Medicine, Community Medical Center, Toms River, NJ, USA; f Department of Emergency Medicine, Beth Israel Deaconess Medical Center, Boston, MA, USA; g Department of Psychiatry and Neurobehavioral Sciences, University of Virginia, Charlottesville, VA, USA

**Keywords:** Trauma, Prehospital care, Health systems, Health policy

## Abstract

**Background:**

In Nigeria, trauma care faces challenges due to high injury and death rates from road traffic accidents and violence. Improvements are underway, but gaps in service availability, training, and coordination persist, necessitating evidence-based interventions.

**Purpose:**

To evaluate trauma care practices in Nigeria, focusing on practitioners' perceptions of training, resources, and care quality to inform policy and practice enhancements.

**Methods:**

An exploratory qualitative study was conducted with seven trauma surgeons across Nigeria, using semi-structured interviews and an Interpretive Description analysis approach, adhering to SRQR standards.

**Results:**

Analysis of interviews with seven Nigerian trauma surgeons highlighted a trauma care system burdened by high incidences of traffic-related injuries. Despite varying caseloads—from 20 cases per month to 65 weekly—common challenges included delayed care, leading to complications like infection and misaligned wound healing. Surgeons noted strengths in motivated staff and sub-specialization but stressed barriers such as underdeveloped prehospital care, financial constraints, and resource shortages, which hindered effective trauma management and outcomes.

**Conclusions:**

Effective trauma care in Nigeria is crucial and achievable through policy reforms, better resource distribution, and enhanced training. Systematic data collection and a national trauma care protocol are recommended to improve patient outcomes and guide future research and policymaking.

**African relevance**

• This study addresses the urgent need for tailored trauma response protocols in Africa's unique healthcare landscape.
• Findings support the adaptation of trauma training to improve surgical outcomes in resource-constrained African settings, as well as for the growth of emergency medicine as a speciality, and prehospital emergency medical service.
• The research provides a foundation for policy development aimed at enhancing trauma care systems across the continent.

## Introduction

Trauma care is a vital component of healthcare systems around the world. It involves the identification, assessment, and management of injuries sustained because of accidents, violence, or other causes of trauma. These injuries can range from minor cuts and bruises to severe injuries requiring surgery and long-term rehabilitation. The goal of trauma care is to prevent death and disability, and to ensure that individuals who have experienced trauma receive the appropriate care and support.

In Nigeria, trauma care is an area of particular concern due to the high rate of injuries and deaths resulting from a range of causes. One of the major contributing factors is road traffic accidents, which are responsible for a significant portion of injuries and deaths in the country. According to the World Health Organization (WHO), Nigeria has one of the highest rates of road traffic deaths in Africa, with more than 39,802 deaths recorded in 2019 [1].

In addition to road traffic accidents, Nigeria also experiences a high incidence of violence, including violence related to terrorism and armed conflict. These and other causes of trauma, such as falls and fires, put a significant strain on the healthcare system and highlight the need for effective trauma care.

Despite the challenges, there are also many efforts underway in Nigeria to improve trauma care. These include the development of specialized trauma centers, which provide advanced diagnostic and treatment services for individuals with severe injuries. There are also initiatives to train healthcare providers in trauma care, including the use of trauma care protocols and guidelines, which outline the steps to be taken in the management of trauma patients.

There are still significant challenges that need to be addressed. One major issue is the inadequate availability of trauma care services, particularly in rural and underserved areas. Many individuals who experience trauma must travel long distances to receive care, which can delay treatment and worsen their prognosis. In addition, the lack of trained personnel and equipment in many healthcare facilities limits the ability to provide effective trauma care.

Another challenge is the lack of coordination and integration among different levels of the healthcare system. Trauma care often requires the involvement of multiple providers and facilities, and the lack of coordination among these can result in fragmented care and poor outcomes.

To address these challenges and improve trauma care in Nigeria, there is need for research on trauma care availability and practice to identify the gaps for targeted intervention. However, there is a paucity of recent, relevant data surrounding trauma practice in the country, rendering it challenging to apply evidence-based interventions and assess the efficacy of policies implemented to improve the quality of trauma care provided. This study aims to evaluate the current situation of trauma practice and the perception of practitioners in the areas of training, resources, and quality of care in Nigeria and bring about downstream policy and practice changes.

## Methods

### Study design and qualitative approach

This exploratory qualitative study aimed to understand the status of trauma care in Nigeria from the viewpoint of trauma surgeons, employing an Interpretive Description approach within a constructivist/interpretivist paradigm. The researchers chose this approach to facilitate a deep understanding of trauma care providers' complex realities and experiences in their specific contexts. The study was conducted between August 2020 and March 2021, with ethical clearance from the National Trauma Hospital, Abuja.

### Researcher characteristics and reflexivity

The research team consisted of Ayobami Olufadeji (AO) and Oluwafunmilayo Akinlade (OA), who have backgrounds in medical research and direct experience with trauma care in Nigeria. Chiamaka Eneh (CE), with expertise in qualitative health research, also contributed to data analysis. The team's diverse knowledge and experiences informed the research design, data collection, and analysis. Researchers practiced reflexivity throughout the study, regularly reflecting on their biases and assumptions and their potential influence on the research process and findings.

### Context

The study was set across all geo-political zones of Nigeria, targeting trauma surgeons working in public healthcare settings. Researchers chose this setting to reflect the diversity of trauma care environments and challenges nationwide.

### Sampling strategy

**Fig. 1:**
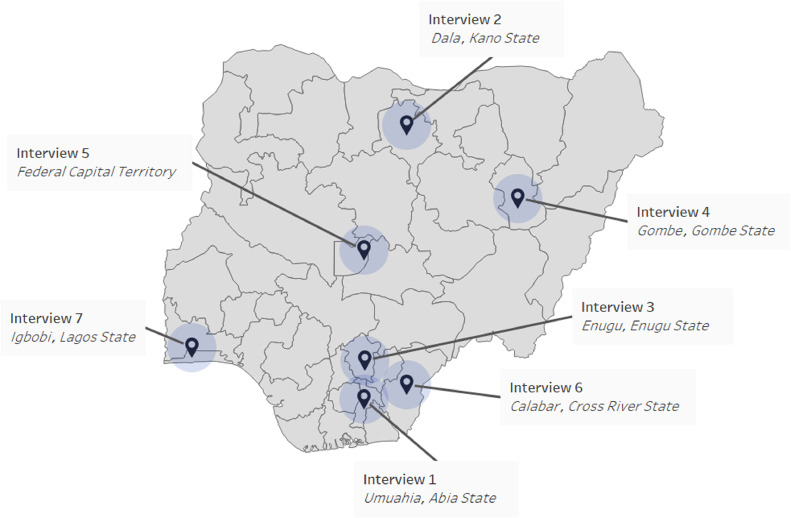
Locations of the interviews.

Participants were purposefully sampled to ensure representation from each geo-political zone in Nigeria, employing criteria focused on years of experience, specialty in trauma surgery, and geographic diversity. Sampling continued until data saturation was reached, with seven trauma surgeons participating (Fig. 1).

### Ethical considerations

Ethical approval was secured from the National Trauma Hospital, Abuja. All participants provided verbal consent, with confidentiality maintained through de-identifying transcripts and using pseudonyms for any illustrative quotes presented.

### Data collection methods

Semi-structured interviews were conducted via teleconference by OA, guided by an interview protocol developed by the research team. The interviews, lasting an average of 45 min, were audio-recorded with consent and transcribed verbatim in English.

### Data collection instruments and processing

A semi-structured interview guide (Supplemental Table 1) was developed and refined through the study to address emerging themes. Teleconferencing and audio-recording devices facilitated the data collection process, with NVivo12 used for data management and analysis. A professional service transcribed Audio recordings verbatim and then uploaded them to NVivo12 for data organization. Transcripts were anonymized, with pseudonyms replacing real names to ensure participant confidentiality.

### Data analysis

An Interpretive Description approach guided the data analysis conducted by OA and CE, focusing on identifying themes and patterns in the participants' descriptions of trauma care experiences. The process included coding, thematic analysis, and reflexivity to minimize methodological bias. Trustworthiness was enhanced through audit trails and consensus discussions among researchers.

### Techniques to enhance trustworthiness

Trustworthiness was ensured by employing several strategies: reflexivity, audit trails, iterative data analysis, and reaching a consensus on divergent codes and themes. These methods underpinned the credibility and reliability of the study findings.

## Results

Seven trauma surgeons from across Nigeria participated, with experiences ranging from 5 to 20 years in trauma care. All were male and represented a variety of public healthcare settings (*Numbers in brackets after quoted statements refer to individual study participants*).

A total of 7 Nigerian trauma surgeons from 7 distinct urban hospitals were analyzed. Each hospital was selected to represent a wide geographic area across Nigeria. The participants’ responses were categorized into three salient themes: (1) the status of trauma care in Nigeria; (2) current barriers to trauma care; and (3) the opportunities for health system improvement. Six of the seven Trauma Surgeons had primary training in Orthopedic Surgery, and one had primary training in Trauma surgery.

### **Status of trauma care in Nigeria**

Participants described the status of current trauma care in Nigeria, including patient characteristics, trauma care within hospitals, outcomes for trauma patients, healthcare personnel, and strengths of the current system.

### Patient profiles

When discussing patient characteristics of trauma cases in Nigeria, participants had similar answers independent of sub-specialization and geographic location. All cited road traffic incidents as the most common cause of trauma, followed by falls, violence including gunshot and stab wounds, burn injuries, assault, and industrial accidents. One participant cited sequelae of delayed trauma care, such as infections, delayed healing, and misaligned bone and wound remodeling as a major issue for patients who did not seek care at the time of initial insult. Trauma caseload varied widely among participants from 20 cases per month to 65 cases per week at a specialized orthopedic spine center in a major city. Patient demographics were one factor that varied depending on the resources of the hospital. For most regional centers, the patient population consisted of those in geographic proximity. This was different for tertiary centers with specialized resources. One participant explained how sub-specialized care and government support attracted patients from all over Nigeria and other parts of Africa to seek care in specialized orthopedic centers: “There are 3 regional orthopedic centers in Nigeria: Lagos, Enugu and Kano that were established by an act by the federal government to provide trauma care only. Recently we've added plastic and reconstructive surgery because we realized that a lot of these patients would need burn [care for their] trauma..... We receive patients from as far as Cameroon, Niger Republic, Chad, Benin Republic, even Mauritania, and Mali” (participant 2).

### Hospital trauma care

All participants were able to describe the typical care pathway for a trauma patient presenting to their health institution. Most facilities had a triage nurse to receive and triage the patient, who was then evaluated by an emergency medicine physician or surgery resident, depending on if the hospital had an emergency medicine department. One surgeon described his institution's protocol: “[We] triage the patient and they are taken straight to the resuscitation room. There, they are attended to using the Advanced Trauma Life Support (ATLS) protocol. They go through the A, B, C and then D, and the patient is stabilized.” Following stabilization and intravenous access, basic labs are ordered, and the patient is assessed on the need for radiographic imaging. He continued, “Once the patient is stabilized, the surgical team on call is invited. If the patient has [an] orthopedic case, trauma or general surgical trauma or if it's pediatric trauma, they'll be invited to see the patient” (Participant 4). Across participant institutions, trauma protocols were based on ATLS, and most participants mentioned obtaining a primary and secondary survey, basic labs, and imaging standard to most trauma workups. However, approaches varied slightly between hospitals. This difference was apparent with the availability of imaging, and whether providers were able to perform X-ray, Focused Assessment with Sonography in Trauma (FAST) exams, and Computed Tomography (CT) scans for patients. Differences also existed in availability of emergency medicine personnel, who when present would stabilize life-threatening medical injuries before transferring care to surgical staff.

Management also varied depending on surgical personnel. At tertiary centers, management was dictated by the type of trauma experienced: “If it's someone who has an abdominal trauma, we have a general surgeon to handle that, someone who has a head trauma, we have a neurosurgeon to handle that…if it's orthopedic trauma, we have a trauma protocol and the patient keys into that protocol and gets treated” (participant 3). For regional, including primary and secondary hospitals, certain cases beyond the capabilities of the hospital which required specialized medical management were referred to tertiary centers following initial stabilization. One physician at a level two trauma center cited the importance of collaboration with such referral centers for the benefit of his patients: “The teaching hospitals and the federal medical centers tend to have more subspecialty services, so we work in collaboration with them to ensure comprehensive health services. I don't think it's about, getting more subspecialties, but making sure that our partnerships and our collaborators are always with us when we need to make such referrals” (participant 7).

### Patient outcomes

Following initial stabilization, referral, or treatment, most participants believed their trauma patients had favorable prognoses. “... probably a large number of them, more than 80, 90 % would still survive once they can get to the hospital” (participant 1). For those who experienced complications, causes varied widely depending on pathology. Some of the most common causes of morbidity and mortality among trauma patients included sepsis, pneumonia, pulmonary embolism, and infection. Orthopedic surgeons attributed their low mortality rates to the nature of injury as nonfatal. “The mortality rate in fractures is not really on the high side. The mortality rate in things like head injuries, chest trauma, abdominal trauma is much higher than what you have for limb fractures” (participant 3). Participants reiterated that most trauma mortality occurred in the prehospital setting, or due to delayed care seeking and late presentation to the hospital.

### Healthcare personnel

Most participants worked in well-resourced tertiary care centers, meaning that health care personnel were some of the most comprehensive among all Nigerian health centers. Subspecialized surgical services across these hospitals included general, trauma, orthopedic, pediatric, plastic, neurologic, cardiothoracic, urologic, oral, and maxillofacial, obstetric and gynecologic surgery services. Specialists reported training in residency programs with specific focuses based on their hospital, including orthopedic and general trauma. Short-term training programs including two-day trauma workshops also existed at specific universities. However, participants reported that a national program had not yet been established or formalized by Nigeria's government to train trauma specialists. Trauma teams worked with a combination of emergency physicians, one to three resident physicians, and an attending (consultant) surgeon always on call, for each subspecialty. Most respondents estimated that approximately five to eight surgeons were available in the hospital at any given time. Certain centers reported the need for recruiting more plastic surgeons to manage complex burns, and cardiothoracic surgeons to manage vascular and thoracic injury patients. Participants also reported variable presence of midlevel staff: “we have nurses trained in accident and emergency and orthopedic care. We also have radiology technicians who work with us to boost capacity of care. We have health attendants on the team, health statisticians and other members of the health care team” (participant 7). Other staff included laboratory technicians and anesthesia technicians, orthotists, health assistants, and physiotherapists. Despite the specialization of care, nearly every participant reported a deficiency of physician and midlevel staff, and that increasing workforce numbers would significantly reduce health care worker burden. Among sampled hospitals that were specifically established to manage trauma cases, participants reported increased knowledge of physician and mid-level staff for handling trauma given their increased caseload and experiential learning.

### Strengths

Many strengths on the status of trauma care in Nigeria were highlighted by participants, including staff motivation and expertise, surgical sub-specialization, and developing training opportunities. Hospital personnel were motivated to care for their patients, and physicians on call were required to be in the hospital at time of work. Reported by one participant, “every specialist is a one-minute call from the accident and emergency room. I think the level of expertise [of our staff] is quite good” (participant 6). Centers which had orthopedic, general, and plastic surgery services mentioned how they could offer comprehensive and multidisciplinary care for complex patients, a benefit not afforded by less resourced institutions. One hospital highlighted the importance of being able to focus solely on trauma cases as a specialized center: “My center is a dedicated trauma building, and we've gone a step further to super specialize in that aspect. So being a dedicated trauma center, and that the West African College of Surgeons have done accreditation and assigned the center a level 1 trauma [designation] by West African standards [is an important factor]. Our trauma care is comprehensive in that we have all specialties on ground” (Participant 5). He continued by highlighting the importance of continuing education for the next generation of trauma surgeons, and how accreditation from a regional governing body supported this aim: “We are accredited for post fellowship in trauma of the West African College of Surgeons. The fellows undergoing training have both academic didactics and hands-on experience. They are taught and have mandatory short courses which they must attend including ATLS, DSTC, RAPTOR. It is comprehensive and that's why we were able to get the accreditation for trauma training. There's no other place in our own geographical location that we see that would provide a higher level of care than we provide” (Participant 5).

### **Barriers**

Despite the strengths of current trauma care in hospital systems in Nigeria, participants named a variety of barriers, weaknesses, and gaps in health care delivery. These gaps included a deficiency of quality, widespread prehospital emergency medicine systems, healthcare financing barriers, healthcare personnel limitations, resource insufficiencies, and interpersonal and cultural barriers.

### Prehospital care

Nearly every participant cited a paucity of prehospital care throughout Nigeria, particularly in non-urban settings. Though an ambulance system existed in Lagos, there was no nationwide standard for prehospital care administration. “Lagos has LASEMA, an agency that tries to do that, tries to offer some care as they are transporting patients” (Participant 1). However, prehospital care was nonexistent outside of the capital city, causing patients to experience delays in receiving care, longer times in transport to the hospital, and increased risk of worsening injury and morbidity: “Most times the prehospital care, especially the ones that happens at the accident scene is not done by qualified personnel. Patients may end up having the injuries even worsened before they come to the hospital. Because most of the well-trained personnel and most of the well-equipped hospitals are in the major cities…that would always result in the prehospital care being substandard” (Participant 3).

### Financial limitations

Most participants listed financial limitations, more specifically poverty, as the greatest barrier to patients receiving adequate trauma care. When discussing the most pressing barriers to care, one physician stated: “The most important one may be patients cannot afford [due to] poverty. There's no health insurance for the patients. Patients are paying out-of-pocket, so even the care that is available, it's difficult for them to access the care that is available” (Participant 1).

The lack of health insurance coverage—universal or private—in Nigeria meant that patients paid completely out of pocket for surgical and medical care, a cost that was expected to be paid at the time of or shortly after care delivery. Some institutions developed an internal financing system, where patients with means would indirectly subsidize the cost of poorer patient's care: “We have a drug revolving fund service. It's just our own little way to circumvent the challenges of patients having to pay from their pockets. It's more or less taking money from the rich and using it to service the needs of the poor in the hospital. When rich people come, we approach [and ask] if they would be willing to put money in that drug revolving fund, which we can draw from to take care of the indigent in society when they present” (Participant 2). Hospitals also reported a deficiency of funding from the Nigerian government, which stalled increasing resource capacity, subsidizing patient care, and educational program development.

### Resource insufficiencies

Every participant listed resource insufficiencies as a barrier to providing adequate diagnostic and therapeutic care for their patients. The most common resource insufficiencies included mechanical ventilators, oxygen tanks, blood for transfusions, and radiographic equipment including CT scans and MRIs. Some facilities lacked ultrasound, thereby preventing providers’ ability to conduct a Focused Assessment with Sonography in Trauma (FAST) exam, a key component of the trauma survey. Even when diagnostic equipment was available, it did not always function: “Sometimes the CT scanner is broken for a few weeks, and sometimes X-ray the same. We don't have an MRI. There are two other facilities outside of the hospital that do, but it's quite expensive” (Participant 6). One participant reported that his hospital had only three mechanical ventilators, despite frequently having more than three patients admitted who could benefit from respiratory support. He and other participants believed increasing mechanical ventilation capacity would decrease mortality from trauma patients in his institution.

### Interpersonal and cultural barriers

A final barrier reported by participants was interpersonal barriers, including patient preference for traditional healers, delayed presentation for care seeking, and health professional burnout. Some patients preferred traditional healers, including bonesetters, to hospital care: “Some believe that traditional bone setters and other alternative health care providers can provide a better care than we do, especially when it has to do with extremities injuries, fractures and all that” (Participant 4). Another physician agreed, explaining that not using the hospital as first-line care led to increased complication rates: “Sometimes, they come having gone to various places, like the traditional bone setters, like a church, and then they come in really bad, or almost near dead. Mortality increases because of late presentation” (Participant 6). Interpersonal barriers also existed from the side of healthcare professionals, including physician burnout. Though reported by only a few participants, they believed a lack of empathy among clinicians led to worse care delivery for patients: “I think some of the issues we have is more of attitude than expertise. I feel like there's no empathy, that the level of empathy required to provide a certain level of care is in short supply… people are not empathetic enough to give their all. People don't know how to be polite and cordial, maybe out of lack of trust for the system generally” (Participant 6). Another physician went as far to characterize what he observed in colleagues as “spiritual warfare,” explaining that the physicians who successfully guarded against a lack of empathy were committed to ideals in their care delivery (Participant 2).

### **Opportunities for health system improvement**

Participants discussed ways to improve trauma care in Nigeria; their strategies centered around improving governmental support and patient outcome data collection and dissemination.

### Government support

Multiple participants mentioned the Nigerian government must provide more financial support and address legislative gaps to improve trauma care in the country. Currently, only two national training associations exist (the West African College of Surgeons and the National Post Graduate Medical College) which have provided quality training to the providers who could receive it. However, other providers had to rely on their hospitals hosting internal training or financially supporting external training in trauma care, and each hospital varied in its willingness and frequency of providing this service. By adding additional government-funded training programs, participants felt it would address the large number of ill-trained physicians and support staff providing trauma care.

Legislatively, government support through public health insurance programs could lead to better health outcomes and reduced financial strain for many patients. “In Nigeria, there's no health insurance so patients pay out of pocket. Most times that hampers service delivery. You know what to do for the patient, but the patient cannot pay. That delays [the patients] care and they may end up developing preventable complications” (Participant 1). Although participants did not explicitly discuss road-safety measures, their consistent identification of road traffic incidents as the leading cause of trauma strongly suggests that governmental initiatives in this area could significantly reduce the number of trauma cases. Potential projects might include the construction of dedicated pedestrian lanes, the institution of laws mandating the use of helmets and seatbelts and improving occupational safety standards in work environments.

### Data gaps

The lack of data collection and dissemination was voiced by participants as a pertinent strategy to improve trauma care. While participants approximated the number or types of traumas they treated monthly or weekly or the associated morbidity and mortality, none could provide confident estimates. No participant mentioned any standardized systems for collecting patient outcome data that could be used for quality improvement or research. Moreover, most participants were unaware of journal articles related to gaps in trauma care or trauma clinical outcomes.

## Discussion

Our research showed striking similarities in perceptions about the state of trauma care, irrespective of the region in which healthcare practitioners operated. This homogeneity suggests a systemic issue in trauma care across Nigeria. Our findings align with those of earlier research, particularly in the prominence of road traffic accidents as a leading cause of trauma care visits. A literature review by Solagberu revealed that 40–90 % of trauma admissions stem from road traffic accidents and violence [2]. Similarly, Thani's systematic review found that 68.4% of trauma admissions were caused by road traffic accidents, followed by falls at 5.5 % [3]. Poor transport infrastructure and lax enforcement of traffic laws were highlighted as significant contributors to the high incidence of these accidents [3].

Our study's findings bear considerable significance for both future research and policymaking. One key observation was the delayed access to specialized trauma care for patients who had to travel long distances, a factor that raises mortality risks and healthcare costs. This observation points toward an under-provision of trauma care facilities, particularly in rural areas. Furthermore, the recent official recognition of emergency medicine as a specialty in Nigeria introduces another layer of complexity. The lack of trained emergency physicians could result in late identification of specific injuries and contribute to inconsistencies in patient care.

Addressing these challenges requires a multi-pronged approach. For future research, focusing on the impact of emergency physicians in trauma care centers would be particularly beneficial, especially given Nigeria's recent initiatives in emergency medicine. On the policy front, there is a pressing need for the development of a national trauma care protocol. A centralized trauma registry could serve as an invaluable tool for data collection, which in turn would inform future policy measures and performance monitoring.

**Fig. 2:**
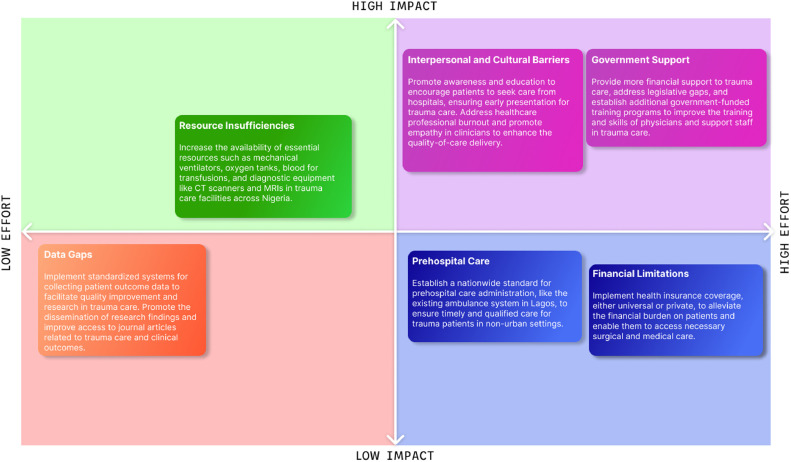
Bridging the gap: evaluating deficiencies and recommended solutions through an impact vs. effort framework.

This study underscores the urgent need for systemic improvements in Nigeria's trauma care landscape. While various challenges exist, from infrastructure to healthcare personnel, the uniformity in these issues across regions indicates opportunities for centralized solutions. Recommendations for policy changes include increased governmental support, improved data collection through a centralized registry, and the establishment of a comprehensive national trauma care protocol. These steps could significantly enhance the effectiveness and efficiency of trauma care services across Nigeria (Fig. 2).

## Limitations

While this study provides valuable insights into the perspectives of trauma surgeons regarding trauma care in Nigeria, there are several limitations that must be acknowledged. First, the study is based on a small sample size of 7 surgeons, which may not fully capture the diversity of experiences and perspectives in trauma care across Nigeria. Second, most participants had primary training in Orthopedic Surgery, potentially introducing a specialization bias into the study findings. Although the hospitals represented were geographically diverse, being limited to urban hospitals may not provide a comprehensive understanding of trauma care challenges and opportunities in rural settings. Furthermore, all participants were from distinct geo-political zones, yet the intricacies within each zone could not be elaborated upon due to the sample size. Lastly, the study focused exclusively on trauma surgeons, thereby omitting the views of other healthcare professionals involved in trauma care, such as emergency physicians, nurses, and first responders, whose perspectives could provide a more holistic understanding of trauma care in Nigeria.

## Conclusion

Overall, the provision of effective trauma care in Nigeria is a critical issue that requires the attention of policymakers, healthcare providers, and other stakeholders. The establishment of emergency medical services will significantly improve the outcomes of trauma patients in Nigeria. In the same vein, efforts directed towards improved in-hospital trauma care are of utmost necessity. More qualitative data on outcomes of trauma patients in the existing facilities will prove useful to policymakers, healthcare providers, and other stakeholders in decision making and policy advocacy. By addressing the challenges and improving access to trauma care services, it is possible to save lives and reduce the burden of injury in the country.

## Dissemination of results

The findings of this research study were shared within the community, from which the data was collected through discussions with the physicians who were interviewed. These discussions allowed for the exchange of insights, clarification of conclusions, and the opportunity to gather feedback directly from the healthcare professionals actively engaged in trauma care in Nigeria.

## Credit authorship contribution statement

**Oluwafunmilayo Akinlade:** Data curation, Methodology, Visualization, Writing – original draft, Writing – review & editing, Project administration. **Adebisi Adeyeye:** Writing – original draft. **Brandon L. Ellsworth:** Formal analysis, Methodology. **Christopher W. Reynolds:** Formal analysis, Methodology. **Chiamaka Eneh:** Data curation. **Ayobami Olufadeji:** Conceptualization, Supervision.

## Declaration of competing interest

The authors declared no conflicts of interest.

## Supplementary materials

Image, application 1
