# Supplementary material for: Exploring trauma surgeons' views on trauma care in Nigeria: A qualitative study
Source: Afr J Emerg Med. 2024 Jul 14;14(4):520–6. doi: 10.1016/j.afjem.2024.03.001 (PMC11731257; doi:10.1016/j.afjem.2024.03.001)
Supplement: Supplementary file 1 [file mmc1.docx]

Interview Questions

1. Can you describe what happens when a trauma patient presents to your hospital for care?
2. Are patients managed using standardized trauma protocol? (Compare the protocol to the checklist)
3. Who provides the trauma care at your hospital?
4. Can you describe the setting where you provide trauma care?
5. What is the physician coverage at your site?
6. How and where are the physicians handling trauma trained to do so?
7. Would you describe the trauma training you provide as comprehensive?
8. What challenges do you face with training?
9. What specialty services are available at your hospital?
10. What specialty services would help you provide more comprehensive trauma care if available?
11. What is your sense of the volume of trauma cases monthly?
12. What are the top 3 causes of the trauma cases you encounter?
13. What challenges do you face with providing adequate trauma care?
14. What factors do you think contribute most to how often you have to transfer patients out to receive care?
15. What is your opinion on the prehospital care your trauma patients receive?
16. What is your sense of the morbidity and mortality of trauma patients at your institution?
17. What do recent journals say about room for improvement in the standard of care?
18. What kind of nursing & mid-level capacity staff do you have as part of the trauma team?
19. What would you say are the strengths of the trauma care provided at your hospital?
20. Is there anything else you feel is essential for me to know?
